# Maternal Serum and Cord Blood Leptin Concentrations in African Newborns: Relationship to Birth Weight and Gender

**DOI:** 10.3390/nu17030515

**Published:** 2025-01-30

**Authors:** Maria Antonietta Castaldi, Salvatore Giovanni Castaldi, Maurizio Guida, Costantino Di Carlo, Laura Sarno

**Affiliations:** 1 Department of Medicine and Surgery, University of Salerno, 84084 Fisciano, SA, Italy; scastaldi@unisa.it; 2 Department of Gynecology and Obstetrics, and Pathophysiology of Human Reproduction, University of Naples “Federico II”, 80138 Naples, NA, Italy; maurizio.guida@unina.it (M.G.); costantino.dicarlo@unina.it (C.D.C.); laura.sarno@unina.it (L.S.)

**Keywords:** leptin, pregnancy, gender, birth weight, Burundi

## Abstract

Background: Leptin, a protein predominantly produced by adipocytes, plays a crucial role in regulating energy balance, inflammation, immunity, and fetal growth. During pregnancy, maternal serum leptin levels increase, peaking in the second trimester, with placental production contributing to this rise. Leptin has also been identified in fetal tissues, and its concentration in umbilical cord blood correlates with birth weight. This study aimed to evaluate serum and umbilical cord blood leptin concentrations in rural Burundian women living in marginal nutritional conditions, and investigate potential gender differences in fetal leptin levels. Methods: We analyzed data from 38 healthy singleton pregnancies (20 male and 18 female newborns) delivered at Hôpital Autonome de Ngozi, Burundi. Leptin concentrations were measured in maternal and umbilical cord blood samples. Results: Our results revealed that neonatal leptin levels were significantly higher in female compared to male newborns, consistent with findings in other populations. Leptin concentrations in umbilical cord blood were positively correlated with neonatal birth weight and the Kaup index, while maternal leptin levels did not show such associations. Conclusions: Despite the challenging nutritional environment in this rural African setting, our findings suggest that leptin’s role in fetal growth regulation may transcend maternal nutritional status. The gender difference observed in leptin levels could be linked to genetic or epigenetic factors rather than fat content or reproductive hormones. This study supports the notion that leptin may be an important regulator of fetal development, even in malnourished populations, and underscores the need for further research to elucidate its mechanisms in pregnancy.

## 1. Introduction

Leptin is a 167-amino-acid protein mainly produced by adipocytes. It acts on the hypothalamic centers, regulating energy expenditure and signaling the amount of body fat mass [1].

Indeed, its serum levels are positively correlated with fat mass, body fat percentage, and body mass index (BMI) [2], and, in adult humans, are further modulated by the dynamic state of energy balance. Leptin also plays a major role in the regulation of different functions, including inflammation, immunity, organogenesis, and reproduction [3,4].

During pregnancy, maternal serum leptin levels progressively increase, peaking in the second trimester and then plateauing to a level at term that is three to four times higher than in nonpregnant women [5]. Furthermore, it has been demonstrated that leptin is produced by the placenta, and placental leptin might contribute to the increase of maternal serum leptin concentration, even though it is not known to what extent [6].

In the mid-90s, it was discovered that leptin is also produced by the placenta and the fetus [7,8]. Indeed, leptin levels in umbilical cord blood significantly increase starting at 34 weeks of pregnancy. A significant correlation exists between the umbilical cord blood leptin concentration and neonatal birth weight. This indicates that leptin may play a role in regulating fetal growth [7,8,9].

Furthermore, it has been demonstrated that female newborns have higher levels of leptin than male newborns [7].

While there is a correlation between leptin levels and birth weight, the exact mechanisms through which leptin influences fetal growth are not yet fully understood [7,8,9]. Specifically, the ways in which leptin affects the development of various fetal organs and tissues remain unclear. Additionally, the functional role of leptin in fetal development—whether it serves merely as a marker or actively regulates growth—requires further exploration. Moreover, the role of leptin in pregnancy-induced metabolic adaptations, as well as the dynamics of placental leptin in physiological and pathological conditions [9,10,11,12], warrants additional investigation.

The aim of this study was to evaluate serum and umbilical cord blood leptin concentrations in the pregnancies of rural Burundian women living a traditional lifestyle, which is characterized by marginal nutritional conditions, and correlate these values with anthropometric parameters. We also explored the gender differences in leptin concentrations in fetuses, a pattern that has previously been observed in adults and children [1,2,13].

## 2. Material and Methods

### 2.1. Setting

This study was set in Ngozi, a town in the north-west of Burundi, Africa.

### 2.2. Study Population

The study populations consisted of 38 newborns (20 males and 18 females) from singleton pregnancies and their 38 rural Burundian mothers who delivered at the obstetrical division of the Hôpital Autonome de Ngozi, Burundi. HIV-positive patients were excluded. The study was conducted in accordance with the Declaration of Helsinki and approved by the local Ethical Committee of the University of Naples “Federico II”, Naples, Italy (actual name: Comitato Etico Campania 3) (code: 558/382; 12/12/2006) for studies involving humans. Informed consent for participation in the study was obtained from all patients.

For each patient, the following anthropometric data were collected: height (3 m/13 mm EC-approved CORECA meter), weight (electronic balance: Charme by Tefal), BMI, and a blood sample, which was drawn from the antecubital vein upon hospitalization. Each sample was centrifuged, tested for HIV (Core HIV 1&2 by Core Diagnostics, Birmingham, UK) and then stored at −20 °C until transport.

For each newborn, the following measurements were obtained: birth weight (SECA 725 pediatric mechanical scale), length (3 m/13 mm EC-approved CORECA meter), and ponderal index. Arterial umbilical cord blood samples were collected at the time of parturition, centrifuged, and stored at −20 °C until transport.

### 2.3. Transport and Storage

All serum samples were transported from Ngozi, Burundi, to our department in Naples, Italy, for radioimmunoassay analysis.

The transport was temperature-controlled at −20 °C and was provided by using GEL PACK U-Teck −20 °C (by PHSE S.r.l., Milan, Italy) and TC30 valid packaging (PHSE S.r.l., Milan, Italy), relying on DHL freight international services. After delivery, all samples were stored at −20 °C until laboratory analysis.

### 2.4. Laboratory Analysis

Serum leptin levels were determined in duplicate with immunoradiometric assays (Human Leptin RIA kit, DRG Instruments GmbH, Marburg, Germany), with a sensitivity of 0.5 ng/mL, an intra-assay coefficient of variation of 3.4–8.3%, and an inter-assay coefficient of variation of 3.0–6.2%.

### 2.5. Statistical Analysis

The statistical analysis of data was performed with the Statistical Package for Social Science 29.0 (SPSS Inc., Chicago, IL, USA).

A two-sided test power calculation was performed in order to detect a difference of 2 ng/mL in leptin levels. The standard deviation was set at 0.25. This power calculation indicated that 31 samples were needed for a power of 80% at a 5% level of significance.

Data distribution was assessed with the Shapiro–Wilk test. All the variables displayed a nonparametric distribution, and a Mann–Whitney U test was used to assess differences between groups.

Correlation between maternal serum and umbilical cord blood leptin levels were regressed on gestational week, neonatal birth weight, Kaup index, and birth weight/birth height, and were evaluated with Spearman’s rho test. Significance was set at *p* < 0.05.

### 2.6. Nutritional Status Assessment

A description of environmental and nutritional conditions was collected, and the typical Burundian lifestyle was inferred by interviewing the 38 women participating in the study. Nutritional data were analyzed using the food composition database for epidemiological studies in Italy [14].

## 3. Results

The study refers to 38 newborns from singleton pregnancies and their 38 mothers, whose characteristics are depicted in Table 1. All of the newborns were healthy, and their mothers had no remarkable illnesses during their pregnancies. The newborns’ gestational age ranged from 38 + 6 to 43 + 1 weeks, and their birth weights ranged from 1700 to 4000 g. The Kaup index (weight in grams divided by the square of height in centimeters, multiplied by 10) ranged from 12.91 to 27.70 (g/cm^2^ × 10). Birth body weight/birth height, which is a good parameter for the fat content in a newborn, ranged from 56.82 to 105.26. The mothers’ ages ranged from 16 to 45 years old, their weights ranged from 35 to 87 kg, and their BMIs ranged from 16 to 45 kg/m^2^.

The serum leptin concentrations were measured in the arterial cord blood of the 38 newborns (20 males and 18 females) and in venous samples of the 38 mothers. All of them had detectable leptin concentrations. Overall, serum leptin concentrations in the newborns ranged from 0.70 to 43.10 ng/mL. Serum leptin concentrations in males (median 7.31 ng/mL, range 0.70 to 31.00 ng/mL) were significantly (*p*= 0.029) lower than those in females (median 13.45 ng/mL, range 2.60 to 43.10 ng/mL). There were no differences in the neonatal birth weight, Kaup index, or birth weight/birth height between male and female newborns.

Serum leptin levels in cord blood were positively associated with neonatal birth weight, Kaup index values, and birth weight/birth height, while maternal serum leptin concentrations were not correlated with these parameters (Table 2).

Additionally, the subjects in the present study were living with environmental and nutritional conditions of starvation [14,15]; indeed, from the in loco-collected data, the principal investigator found out that the typical Burundian lifestyle consists of one meal a day composed of 100 g of rice or potatoes or bananas, 50 g of tomatoes or lenga-lenga (*Amaranthus dubius*), and one mango or one papaya, for a total of 1640.4 Kcal versus the 2489 Kcal daily calories recommended by western feeding standards for pregnant women [15,16] (Supplementary Table S1).

## 4. Discussion

Since its discovery in 1994, leptin has been linked to BMI, with an exponential relationship observed in adults, suggesting that fat mass is the primary regulator of leptin levels [1]. However, there is also a clear dependence upon age and pubertal stage, since there is a decrease in males with age compared to females [13]. Nevertheless, although the relative increase of leptin vs. BMI was identical in both genders, leptin levels increased by the same factor, absolute values were significantly different, suggesting that an additional factor influences plasma leptin [13].

Furthermore, in pregnancy, there are increased leptin levels observed starting in the first trimester; this increase is known to precede the weight gain typical of the second and third trimesters. Therefore, as the role of leptin in pregnancy is unclear, though it is supposed to act as a growth factor, the significance of this increase remains unexplained, particularly since the increase does not cause either a reduction in appetite or an augmentation in energetic expenditure [5,17,18].

Additionally, it has been demonstrated that leptin values at birth correlate with birth weight and Kaup index values, thus suggesting a function of leptin in the control of adipostat in the uterus, even though fetuses do not need to control their own but, rather, completely depend on trans-placental uptake for their energy supply [5].

Therefore, our results are perfectly in accord with these data that suggest the possible role of leptin in the control of adipostat, even in a malnourished population such as this Burundian one. Indeed, a recent study showed that prenatal lipid-based nutrient supplements increased cord leptin concentrations in pregnant women from rural Burkina Faso [19].

Moreover, there are interesting discrepancies between maternal and cord blood serum leptin values in physiological pregnancies and pathological ones: in diabetic women (either type 1 or gestational diabetes) maternal serum leptin levels are like those found in normal pregnancies, while fetal levels are higher in diabetic pregnancies than those in physiological pregnancies [5,20].

Eventually, in pregnancies with intrauterine growth restriction (IUGR), cord blood leptin values are markedly reduced, while maternal levels are increased; this very fact could be due to the low adipose mass of IUGR fetuses, while maternal hyperleptinemia could be explained by a mechanism of placental compensation [9,21]. Indeed, during pregnancy, one of the greatest producers of leptin is the placenta, which introduces 95% of its produced leptin into maternal circulation and only 5% into fetal circulation [9].

Thus, our data are consistent with the fact that, according to international literature, arterial umbilical cord blood leptin is both produced by and pertinent to the fetus [17,18]. Furthermore, the positive correlation between umbilical cord leptin levels and neonatal birth weight/Kaup index values supports the role of leptin in regulating fetal growth. Therefore, measuring leptin in cord blood provides an accurate method for assessing fetal leptin levels.

Our study is the first to show that, in a starved African population, cord blood leptin concentrations were correlated with neonatal anthropometric parameters, and that female newborns had higher levels of leptin than male newborns.

Actually, malnourished individuals from rural Africa are rarely included in leptin studies [22], thus providing important new information on the regulation of fetal growth in malnourished environments. Indeed, our data are consistent with previous studies [5,6,7,8,9] that demonstrate similar patterns of leptin behavior in western populations.

Therefore, this gender difference, shown by our data, in the fetus is unlikely to be due to either body fat content, its distribution, or to reproductive hormone status, in particular E2 and testosterone, which have been demonstrated to have similar concentrations in male and female newborns [7]. Consequently, our data strongly support the fact that the existence of a gender difference, even in the fetus of a malnourished mother, may depend on genetic differences or the epigenetic regulation of the *ob* gene. Indeed, it has been recently reported in mice that female mice have a different profile of DNMT expression in both wild-type and those with an obese/diabetic condition, leading to the development of metabolic disease and changes in kidney morphophysiology [23]. Indeed, a recent mouse study found that low-dose maternal exposure to BDE-47 led to weight gain in female offspring and impaired glucose and insulin tolerance in both sexes. In vitro and in vivo data indicate that BDE-47 may promote increased adipogenesis, potentially through DNA hypermethylation. Additionally, mRNA analysis suggests that disrupted leptin signaling resulting in neuronal dysregulation of energy homeostasis could contribute to weight gain and impaired insulin and glucose tolerance [24].

Our findings contribute to the growing body of evidence highlighting sex-specific factors in prenatal development, emphasizing significant gender differences in neonatal leptin levels.

Furthermore, we can assess that, in the present African population of Burundian women and newborns, leptin could have a significance comparable to western populations, even if these populations are very different. Indeed, Burundian mothers are leaner [5,6,7,8,9] and have a very different nutritional status than western ones, and moreover, Burundian newborns have a median birth weight that is physiological, but at the lower limit of European standards [11].

A potential limitation of this study is its small sample size, consisting of only 38 women. However, the results align perfectly with the power analysis calculations presented in the statistics section. Further studies with larger sample sizes would be necessary to confirm the present findings and clarify the role of leptin in pregnancy.

The findings from this research could be further explored in various ways; here, we suggest three possible directions. First, monitoring maternal leptin patterns over time and in newborns might shed light on whether variations in growth, metabolism, or health outcomes later in life are associated with greater leptin levels in females. To support causal inferences, consider other maternal factors that may affect leptin concentrations, such as BMI, inflammation, or metabolic status, including pregnancy-induced metabolic adaptations. Second, comparing leptin levels and their correlations in well-nourished populations—both in physiological pregnancies and those complicated by conditions such as gestational diabetes, hypertension, IUGR, and obesity—could enhance our understanding of how malnutrition may affect leptin’s function. A third direction for future research could focus on the genetic expression and epigenetic regulation of leptin during fetal development. Recent studies involving mother–child pairs from a birth cohort have revealed a strong association between DNA methylation age (DNAmAge) and leptin [25], suggesting that epigenetic factors may play a critical role in regulating leptin levels and influencing fetal growth.

In conclusion, despite significant advances in modern technology, which now allow for the detection of cancer biomarkers even during prenatal testing [26], many critical issues regarding fetal development during pregnancy remain unresolved. A comprehensive understanding of the complex molecular mechanisms that govern fetal growth and development is still needed to fully harness their potential in clinical practice. Hence, leptin could play a role in controlling fetal growth, although its influence may not be highly dependent on maternal nutrition status.

Indeed, the correlation between umbilical cord leptin concentrations and neonatal birth weight, the Kaup index, and the birth weight-to-height ratio indicates that leptin may have a role in regulating fetal growth. This finding is consistent with the idea that leptin helps regulate energy balance and growth, even during pregnancy.

## Figures and Tables

**Table 1:** Characteristics of subjects and serum leptin in cord and maternal blood.

	Total (*n* = 38) Median and Range	Male (*n* = 20) Median and Range	Female (*n* = 18) Median and Range
Characteristics of newborns			
Gestational Age	40 + 0 [33 + 0–42 + 6]	40 + 0 [33 + 0–43 + 1]	40 + 0 [33 + 0–42 + 6]
Birth body weight (g)	2950 [1700–4000]	2837 [2300–3700]	3200 [2500–4000]
Kaup index (g/cm^2^ × 10)	20.77 [12.91–27.70]	20.45 [15.12–25.62]	22.05 [14.69–27.70]
Birth body weight/birth height	78.94 [56.82–105.26]	75.98 [58.97–97.37]	84.21 [56.82–105.26]
Characteristics of mothers			
Body weight (kg)	55.38 [35.00–87.00]	55.50 [43.00–83.00]	55.00 [35.00–87.00]
Body mass index (BMI kg/m^2^)	22.28 [17.86–31.95]	22.33 [19.48–31.32]	22.23 [17.86–31.95]
Serum leptin levels			
Maternal serum leptin	4.80 [2.00–40.70]	7.20 [2.00–35.90]	8.36 [2.00–40.70]
Cord blood leptin	6.84 [0.70–43.10]	7.31 [0.70–31.00]	13.45 [2.60–43.10] *

* *p* = 0.029 vs. male.

**Table 2:** Correlations between maternal serum and cord blood leptin concentrations with various parameters for each group: total, male, and female (Spearman’s rho).

		Total (*n* = 38)		Male (*n* = 20)		Female (*n* = 18)	
		r	*p*	r	*p*	r	*p*
Cord Blood Leptin	Gestational week	0.282 (*)	0.048	0.381 (*)	0.049	0.436 (*)	0.046
	Birth body weight (g)	0.449 (**)	0.003	0.412 (*)	0.036	0.436 (*)	0.046
	Kaup index (g/cm^2^ × 10)	0.453 (**)	0.003	0.417 (*)	0.034	0.441 (*)	0.044
	Birth body weight/birth height	0.453 (**)	0.003	0.417 (*)	0.034	0.441 (*)	0.044
	Body weight (kg)	0.334 (*)	0.023	0.410 (*)	0.036	0.402	0.061
	Body mass index (BMI kg/m^2^)	0.305 (*)	0.035	0.302	0.098	0.433 (*)	0.047
Maternal Serum Leptin	Gestational week	−0.189	0.134	−0.309	0.093	0.039	0.442
	Birth body weight (g)	0.289	0.051	0.202	0.197	0.361	0.085
	Kaup index (g/cm^2^ × 10)	0.290	0.059	0.206	0.192	0.356	0.088
	Birth body weight/birth height	0.290	0.059	0.206	0.192	0.356	0.088
	Body weight (kg)	0.481 (**)	0.001	0.425 (**)	0. 001	0.579 (**)	0.009
	Body mass index (BMI kg/m^2^)	0.571 (**)	0.001	0.521 (**)	0.009	0.716 (**)	0.001

* *p* < 0.05. ** *p* < 0.01.

## Data Availability

The data that support the findings of this study are available on request from the corresponding author, M.A.C. The data are not publicly available due to protected health information.

## Supplementary Materials

The following supporting information can be downloaded at: https://www.mdpi.com/article/10.3390/nu17030515/s1, Table S1: Typical Burundian foods and their nutritional composition.
