# Supplementary material for: Maternal Serum and Cord Blood Leptin Concentrations in African Newborns: Relationship to Birth Weight and Gender
Source: Nutrients. 2025 Jan 30;17(3):515. doi: 10.3390/nu17030515 (PMC11821249; doi:10.3390/nu17030515)
Supplement: Supplementary file 1 [file nutrients-17-00515-s001.zip › nutrients-3422119-supplementary.pdf]

Table S1. Typical Burundian foods and their nutritional composition [14].

Burundian Typical Lifestyle consists in 1 meal a day composed by: 100 g of rice or potatoes or bananas, 50 g of tomatoes or lenga – lenga, and 1 Mango or 1 Papaya, for a total of 1640,4 Kcal versus 2489 Kcal daily calories given by western feeding standards for pregnant women [15, 16]				
	Kilocalories per 100g	Carbohydrates in 100g	Proteins in 100g	Lipids in 100g
Rice	362 Kcal	87,6g	7g	0,6g
Potatoes	85 Kcal	18g	2,1g	1g
Bananas	66 Kcal	15,5g	1,2g	0,3g
Tomatoes	17 Kcal	2,8g	1,2g	0,2g
Lenga – lenga (*Amaranthus dubius*)	31 Kcal	3g	3,4g	0,7g
Mango	57 Kcal	14,1g	0,7g	0,2g
Papaya	36 Kcal	8,8g	0,5g	0,1
